# Diagnostic and therapy of an early‐stage primary leiomyosarcoma of the sigmoid colon: A case report

**DOI:** 10.1002/ccr3.9178

**Published:** 2024-07-12

**Authors:** M. Fickenscher, N. Gvozdenovic, O. Ponsel

**Affiliations:** ^1^ Department of General and Visceral Surgery Klinikum Bayreuth Bayreuth Germany

**Keywords:** colon, colonic, diagnostic, leiomyosarcoma, surgical resection, therapy

## Abstract

**Key Clinical Message:**

Primary leiomyosarcoma of the colon is a very rare tumor entity. Because of unspecific findings, diagnostic can be challenging. Most cases are diagnosed in advanced stages with poor overall survival. Unclear histological findings of smooth muscle cell tissue in colon biopsies together with a tumor of the colon wall in computed tomography (CT) imaging should lead to the differential diagnosis of primary colonic leiomyosarcoma and further diagnostic procedures.

**Abstract:**

Primary colonic leiomyosarcoma is an extremely rare tumor entity arising from smooth muscle cells in the colon wall. Only 0.1% of all colorectal malignancies are leiomyosarcomas. Most patients are diagnosed in advanced disease stages. The overall survival rates are low, and recurrence rates are high. Only few data regarding the outcome in localized early disease stages are available. We report the case of an early‐stage primary leiomyosarcoma of the sigmoid colon treated with surgical resection. We present the case of a 53‐year‐old male patient who underwent a colonoscopy due to intermittent rectal bleeding. Colonoscopy revealed an intraluminal polypoid growing tumor in the sigmoid colon. A biopsy was performed with inconclusive histological results. A CT scan revealed a process in the sigmoid colon with wall thickening; there was no evidence of metastatic lesions. After laparoscopic oncological resection of the sigmoid colon, histological examination surprisingly revealed a localized high‐grade leiomyosarcoma. Primary leiomyosarcoma of the colon is a rare tumor entity and diagnostic can be challenging. Only a few patients with colonic leiomyosarcoma diagnosed in localized early disease stages and treated with complete surgical resection have been reported in the literature. These patients seem to have a better prognosis with longer overall survival. Because of unspecific diagnostic findings and the lack of symptoms in early disease stages, interdisciplinary collaborations between gastroenterologists, radiologists, pathologists, and surgeons are crucial for early diagnosis and treatment.

## Graphical abstract

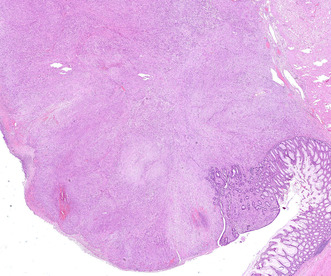

## 1 INTRODUCTION

Leiomyosarcoma (LMS) of the gastrointestinal tract is a rare tumor entity arising from smooth muscle cells in the bowel wall.^1^ LMSs are associated with specific genetic abnormalities and risk factors such as radiation exposure and immunodeficiency.^2^ The mean age at diagnosis is between the fifth and sixth decades of life with no female or male predominance.^3^, ^4^ The main localizations of gastrointestinal LMSs are the small intestine and, less commonly, the colon, and rectum.^4^ Primary colonic LMS (pcLMS) is an extremely rare tumor that accounts for only 0.1% of all colorectal malignancies.^5^

Because of nonspecific symptoms, diagnostic of pcLMS can be challenging and is often delayed. Most cases are diagnosed as incidental findings or in advanced disease stages. Only a few cases, especially in early localized stages, have been reported in the literature. The largest available literature review from Bananzadeh et al. contains 36 cases of pcLMS.^6^

In this article, we report the rare case of a 53‐year‐old male with an early‐stage primary LMS of the sigmoid colon treated with laparoscopic resection.

## 2 CASE HISTORY/EXAMINATION

A 53‐year‐old Caucasian male patient underwent an outpatient colonoscopy due to intermittent rectal bleeding. Fever, weight loss, night sweats, and increased infection susceptibility were denied. The family history of cancer was negative. The physical examination of the abdomen was unremarkable. Body mass index (BMI) 27 kg/m^2^. Tumor markers, including carcinoembryonic antigen (CEA) and carbohydrate antigen 19–9 (CA 19–9), were within normal levels. Colonoscopy revealed an intraluminal polypoid growing tumor in the sigmoid colon (Figure 3). A biopsy was performed; however, there was no histological evidence of malignancy. An abdominal computed tomography (CT) scan revealed a process in the sigmoid colon with moderate wall thickening, which was suspicious for invagination of the sigmoid colon (Figure 4). There was no evidence of lymph node metastasis or other metastatic lesions.

## 3 METHODS (DIFFERENTIAL DIAGNOSIS, INVESTIGATIONS, AND TREATMENT)

A repeat colonoscopy was performed in our clinic, and a biopsy then revealed an inflammatory pseudotumor with smooth muscle cell tissue. Endoscopic appearance, imaging, and histological examination considered, differential diagnoses of this colonic mass included different malignancies as well as benign diseases (e.g., intestinal leiomyoma, intussusception).

The case was discussed in our multidisciplinary tumor conference. Due to suspicion of malignancy, surgical resection was recommended. A laparoscopic resection of the sigmoid colon was performed according to oncological criteria.

## 4 OUTCOME AND FOLLOW‐UP

The postoperative course was uneventful, and the patient was discharged on time.

**FIGURE 1:**
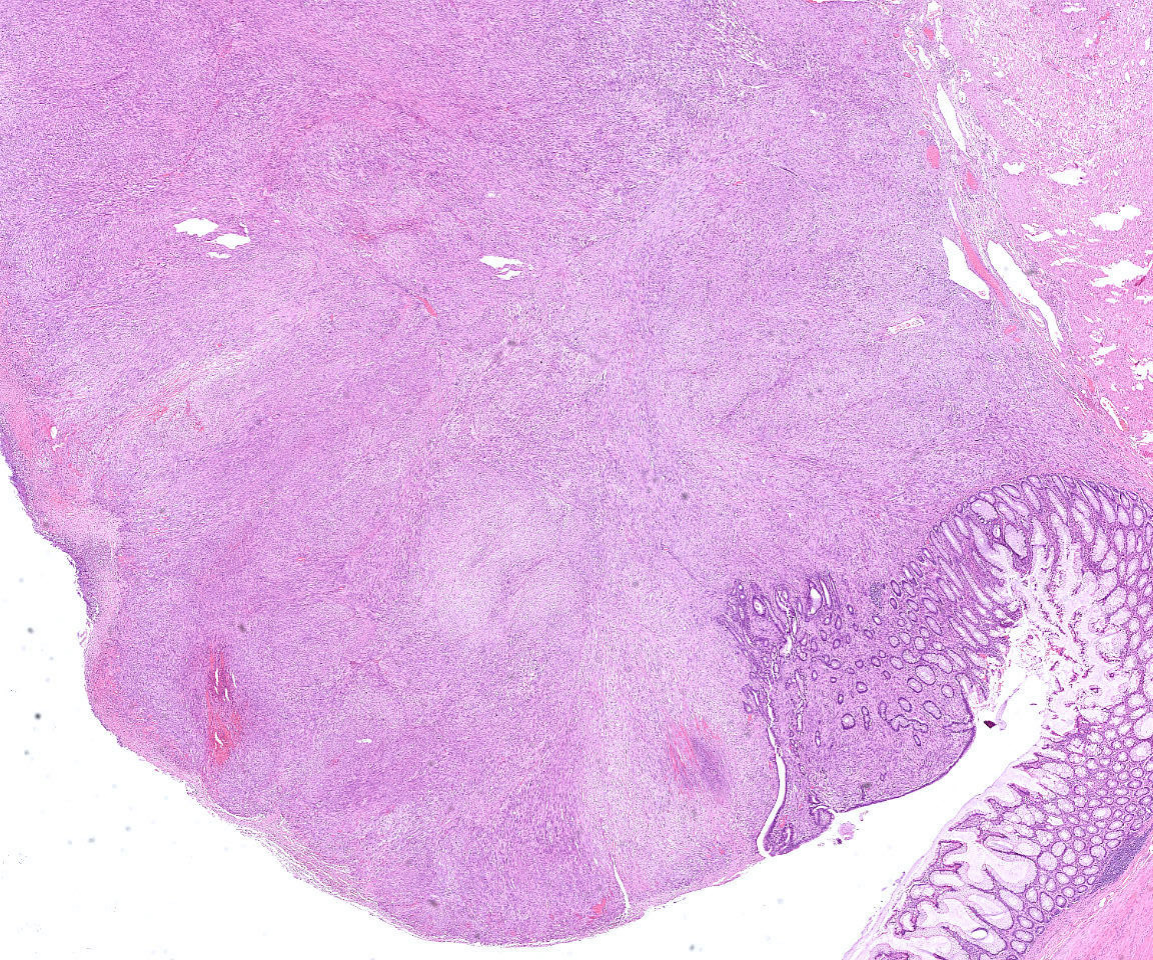
Histological image showing a colonic leiomyosarcoma with mucosal infiltration.

**FIGURE 2:**
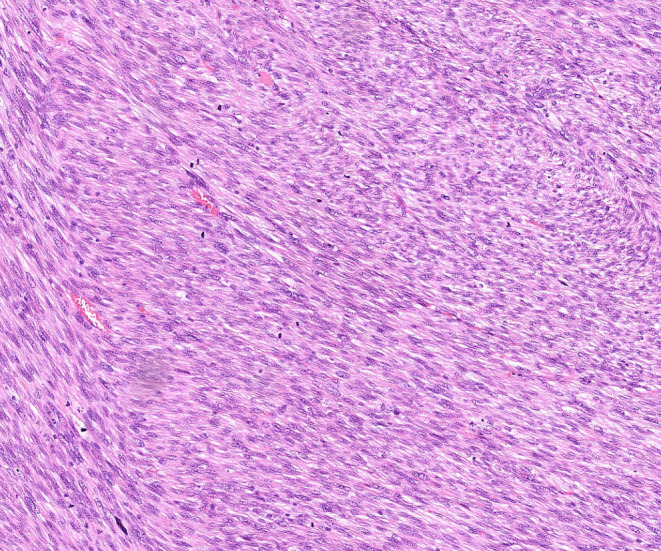
Microscopic appearance of a colonic leiomyosarcoma. Tumor cells have spindle‐shaped nuclei with eosinophilic cytoplasm.

Histopathological examination revealed a high‐grade leiomyosarcoma of the sigmoid colon (pT1, L0, V0, Pn0, pN0 (0/9), R0), tumor grade G3 (Table 1). The tumor dimensions were 4.2 × 4.1 × 2.8 cm. Histologically, there were partially polymorphic spindle cells and foam cells with vimentin detection (Figures 1 and 2). Immunohistochemical analysis of the tumor showed negativity for DOG1 and CD34. The Ki67 index was 25%, mitotic activity 50/10 HPF, and tumor necrosis was approximately 10%. There was no regional lymph node metastasis.

**TABLE 1:** Fédération Nationale des Centres de Lutte Contre le Cancer (FNCLCC) Histologic Grading System for soft tissue sarcomas.^19^

FNCLCC Histologic Grading System	
Tumor differentiation	
Score 1	Closely resembling normal tissue
Score 2	Histological typing is certain
Score 3	Embryonal or undifferentiated sarcomas
Mitotic count	
Score 1	0–9 mitoses per 10 HPF
Score 2	10–19 mitoses per 10 HPF
Score 3	>19 mitoses per 10 HPF
Tumor necrosis	
Score 0	No necrosis
Score 1	<50% tumor necrosis
Score 2	>50% tumor necrosis
Histological grade	*Grade 1*: total score 2–3, *Grade 2*: total score 4–5, *Grade 3*: total score 6–8

**FIGURE 3:**
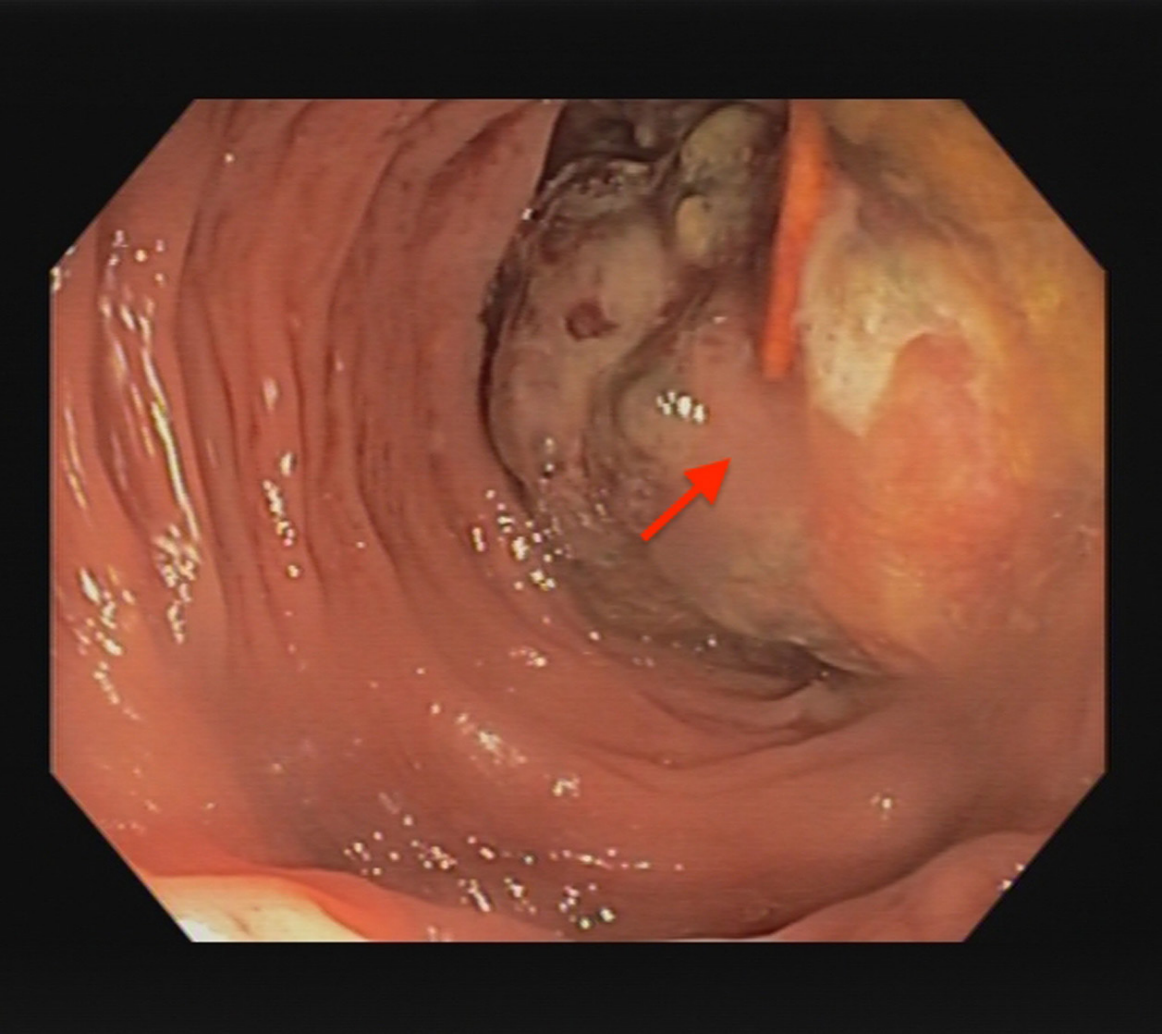
Colonoscopy with an intraluminal polypoid growing leiomyosarcoma of the sigmoid colon.

**FIGURE 4:**
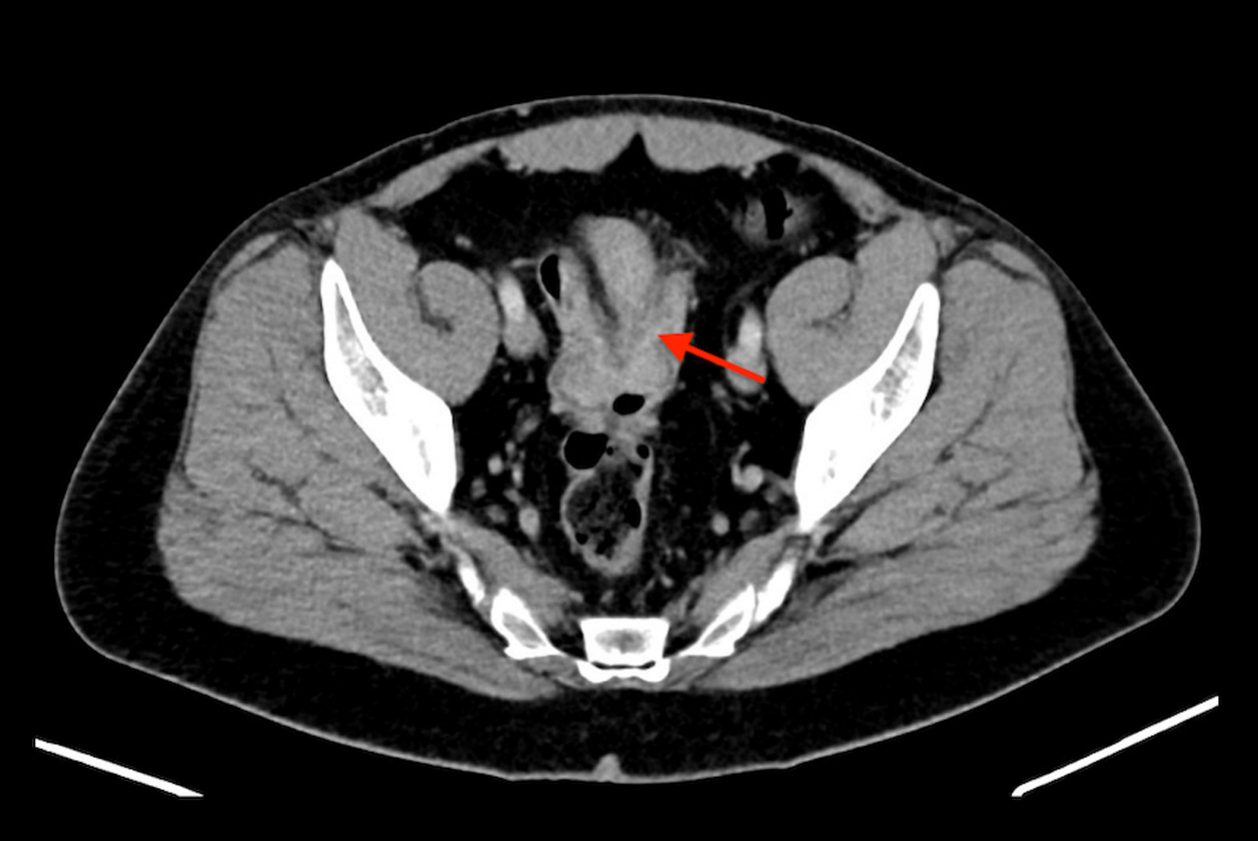
Abdominal computed tomography of the patient showing a tumor mass of the sigmoid colon.

The patient was referred to a specialized sarcoma center. Due to the tumor grade G3, the multidisciplinary tumor conference recommended adjuvant chemotherapy with doxorubicin/dacarbazine, and if applicable, in combination with regional hyperthermia.

## 5 DISCUSSION

Diagnostic of pcLMS is challenging. The diagnosis of pcLMS is often delayed, especially in the early disease stages. The clinical symptoms are nonspecific and vary greatly from weight loss and abdominal pain to stool irregularities and gastrointestinal bleeding. Clinical signs may be missing, especially in localized early disease stages.^6^ To date, no biomarkers have been established for diagnostic blood tests.^7^ Further diagnostics with ultrasound, CT scan, and magnetic resonance imaging (MRI) allow the detection of a tumor mass and may help to assess the tumor extension, but imaging features are frequently nonspecific regarding the tumor entity. On CT scan, a pcLMS appears as a hypodense mass with homogeneous (in early disease stages) or heterogeneous (in advanced disease stages with central necrosis or calcification) enhancement.^8^ Differential diagnostic considerations in CT imaging include other malignancies (e.g., adenocarcinoma, gastrointestinal stromal tumor (GIST), lymphoma, neuroendocrine tumor (NET)), and benign diseases (e.g., intestinal leiomyoma, intussusception).^9^, ^10^

A histopathological examination is required to confirm the diagnosis of pcLMS. Biopsy is mainly performed during colonoscopy. The colonic mucosa may appear inconspicuous because of the exophytic growth from the smooth muscle layer of the colon wall. The typical endoscopic features of malignancies are frequently missing.^11^ A targeted biopsy can be challenging, and unclear histological findings may lead to delayed diagnosis. Immunohistochemical analysis is necessary for further differentiation and exclusion of other mesenchymal tumors. LMS is characterized by positivity for desmin, vimentin, alpha‐smooth muscle actin, and h‐caldesmon.^12^ KIT, CD34, CD117, or DOG1 negativity helps to exclude GISTs.^13^ In most cases, pcLMS is localized in the sigmoid colon, followed by the ascending colon.^6^

Because of nonspecific clinical symptoms and diagnostic signs, an interdisciplinary collaboration between gastroenterologists, radiologists, pathologists, and surgeons is crucial for diagnosing pcLMS in early disease stages. Unclear histological findings of smooth muscle cell tissue in colonic biopsies and a tumor of the colon wall on CT imaging should lead to the differential diagnosis of pcLMS and further diagnostics.

In localized stages, surgical therapy for patients with pcLMS is performed as oncological colon resection. A laparoscopic approach should be pursued for patients with moderate tumor size.^14^ In locally advanced tumors, an open surgical resection should be considered.^14^ The aim is to achieve a total tumor resection (R0). Wang et al. showed that the postoperative outcome of patients with pcLMS is negatively influenced by tumor size (>8 cm) and patient age (>60 years).^15^ The available data regarding the correlation between mitotic counts and overall survival is unclear.

After surgical resection, adjuvant chemotherapy is recommended for patients with high‐risk gastrointestinal LMS.^16^, ^17^ High‐risk LMS was defined as tumor size >5 cm and high tumor grade (G2/G3, according to the FNCLCC Histologic Grading System, Table 1).^1^, ^13^, ^17^ In special risk constellations, a combination of adjuvant chemotherapy with regional hyperthermia should be considered.^17^

Due to the small number of cases, only limited data are available regarding the outcomes of patients with colonic LMS after surgical resection in localized disease stages. However, patients with tumors limited to the mucosa and submucosa seem to have better prognoses and longer survival.^1^

pcLMS predominantly metastasizes to the liver and lung.^1^, ^18^ Lymphogenous spread occurs rarely.^18^ In patients with metastatic disease or locally advanced inoperable gastrointestinal LMS, palliative systemic chemotherapy is recommended.^16^ Resection of pcLMS metastases has rarely been described.^18^ The median survival of patients with metastatic pcLMS is very limited. A study by Yamamoto et al. with 11 patients, reported a 5‐year overall survival of 9.1%.^1^

## 6 CONCLUSION

Primary LMS of the colon is a rare disease and diagnostic can be challenging. Interdisciplinary collaboration between gastroenterologists, radiologists, pathologists, and surgeons is crucial for early diagnosis. Overall survival of patients diagnosed in advanced stages is very limited. Only patients who receive early diagnosis and treatment seem to have a better overall survival. Because of the rarity of these tumors, there are little available data regarding risk factors and outcomes. Prospective multicenter studies are needed to evaluate treatment options and long‐term outcomes in different disease stages.

## AUTHOR CONTRIBUTIONS

**M. Fickenscher:** Conceptualization; data curation; formal analysis; investigation; methodology; validation; visualization; writing – original draft; writing – review and editing. **N. Gvozdenovic:** Conceptualization; data curation; investigation; writing – original draft. **O. Ponsel:** Project administration; resources; supervision; validation.

## FUNDING INFORMATION

This research received no external funding.

## CONFLICT OF INTEREST STATEMENT

The authors declare no conflict of interest.

## ETHICS STATEMENT

The authors declare that human ethics approval was not needed for this study.

## CONSENT

Written informed consent was obtained from the patient to publish this report in accordance with the journal's patient consent policy.

## Data Availability

The authors of this manuscript are willing to provide additional information regarding the case report.
